# Effects of Isoflavone-Enriched Feed on the Rumen Microbiota in Dairy Cows

**DOI:** 10.1371/journal.pone.0154642

**Published:** 2016-04-28

**Authors:** Jitka Kasparovska, Martina Pecinkova, Katerina Dadakova, Ludmila Krizova, Sylvie Hadrova, Matej Lexa, Jan Lochman, Tomas Kasparovsky

**Affiliations:** 1 Department of Biochemistry, Faculty of Science, Masaryk University, Brno, Czech Republic; 2 Department of Information Technologies, Faculty of Informatics, Masaryk University, Brno, Czech Republic; College of Agricultural Sciences, UNITED STATES

## Abstract

In this study, we compared the effects of two diets containing different isoflavone concentrations on the isoflavone transfer from feed into milk and on the rumen microbiota in lactating dairy cows. The on-farm experiment was conducted on twelve lactating Czech Fleckvieh x Holstein cows divided into two groups, each with similar mean milk yield. Twice daily, cows were individually fed a diet based on maize silage, meadow hay and supplemental mixture. Control group (CTRL) received the basal diet while the experimental group (EXP) received the basal diet supplemented with 40% soybean isoflavone extract. The average daily isoflavone intake in the EXP group (16 g/day) was twice as high as that in the CTRL group (8.4 g/day, P<0.001). Total isoflavone concentrations in milk from the CTRL and EXP groups were 96.89 and 276.07 μg/L, respectively (P<0.001). Equol concentrations in milk increased from 77.78 μg/L in the CTRL group to 186.30 μg/L in the EXP group (P<0.001). The V3-4 region of bacterial 16S rRNA genes was used for metagenomic analysis of the rumen microbiome. The experimental cows exhibited fewer OTUs at a distance level of 0.03 compared to control cows (P<0.05) and reduced microbial richness compared to control cows based on the calculated Inverse Simpson and Shannon indices. Non-metric multidimensional scaling analysis showed that the major contributor to separation between the experimental and control groups were changes in the representation of bacteria belonging to the phyla *Bacteroidetes*, *Proteobacteria*, *Firmicutes*, and *Planctomycetes*. Surprisingly, a statistically significant positive correlation was found only between isoflavones and the phyla *Burkholderiales* (r = 0.65, P<0.05) and unclassified *Betaproteobacteria* (r = 0.58, P<0.05). Previous mouse and human studies of isoflavone effects on the composition of gastrointestinal microbial populations generally report similar findings.

## Introduction

The health and productivity of cows are highly dependent on the composition of their diet [1]. The rumen is a highly diverse ecosystem comprising three taxonomic groups of microorganisms: bacteria, protozoa, and fungi, which are active in degrading or utilising different feed components: cellulose, hemicellulose, intermediate acids, sugars, starch, proteins, and lipids [2–4]. Bacteria comprise the majority of rumen microorganisms (10^10^ to 10^11^ cells/ml of rumen content) [4]. They convert feedstuffs to short-chain fatty acids and microbial proteins [5]. Most of the bacteria in the rumen are obligate anaerobes which are sensitive to oxygen [2]. Bacterial populations in the rumen can be influenced by various factors, such as diets, species and age of hosts, feeds, feed additives, seasons, and geographic regions [4, 6]. Furthemore, the metabolic activity of ruminal anaerobic bacteria is influenced by changes in pH and redox potential values [7, 8].

Phytoestrogens are polyphenolic, nonsteroidal, secondary plant metabolites that possess estrogenic properties. They may also posess beneficial effects as estrogen agonists or antiestrogens in human health and disease. The health benefits of phytoestrogens are useful for cardiovascular disease, hormone-dependent cancers (particularly breast and prostate cancer), osteoporosis, menopausal symptoms [9, 10], obesity, and type-2 diabetes [11]. Isoflavones with the most potent estrogenic activity belong to a class of phytoestrogens, whereas major isoflavones were detected in leguminoses [12]. The clinical effectiveness of soy or isoflavone-rich products is believed to be dependent on the ability of the product to metabolize from daidzein (a product component) to equol. However, only approximately 30–40% of humans can produce equol (so called equol-producers [13]). Because equol is in vitro more bio-active than its precursor daidzein: it has a higher oestrogenicity [14, 15], it is a more potent anti-oxidant [16] and it possesses anti-androgenic properties [17], the ability of humans to produce equol could, at least, partly explain the differences in the biological effects observed following soy consumption. Thus, an oral administration of equol seems to be an alternative strategy for obtaining the health-promoting benefits of this substance in non-equol producers [13]. Equol, as a predominant bacterial metabolite of isoflavones, can be found at various concentrations in some foods of animal origin, especially in cow´s milk where the rate of equol excretion depends largely on metabolism in the rumen which is tightly connected to bacterial composition. Recent studies suggest [18, 19] that bovine milk and some dairy products can be considered as a potential dietary source of equol for non-equol producing human. In fact, milk produced by dairy cows fed fresh or ensiled red clover can contain up to several hundred micrograms of equol per litre [20].

Two major sources of phytoestrogens are commonly used to nourish lactating dairy cows. One source, red clover (*Trifolium pratense*), is rich in isoflavones formononetin, biochanin A, and prunetin, and is weakly estrogenic in cows. The other source, soy (*Glycine max*. L. Merr.), contains mainly daidzein, genistein, and glycitein [20–22]. After oral ingestion by ruminants, the glycosides are hydrolyzed by bacterial glucosidases to aglycones. Further, aglycones are highly metabolized by rumen microorganisms [23] that convert daidzein into equol [24] and genistein into inactive metabolite p-ethyl-phenol [13].

Although microbial diversity in the rumen depends on the diet composition of the ruminants [6], studies focused on isolating and identifying the bovine rumen bacteria that convert daidzein to equol are scarce [25, 26]. According to Edwards et al. [27], the rumen may contain 300–400 bacterial species. Characterizing the ruminal microbiome, particularly the bacterial populations, by phylogenetic analysis of 16S rRNA gene sequences recovered from clone libraries by direct PCR amplification [27, 28] is interesting because the characterization is essential for functional analysis [29]. This characterization could be an underestimation as only a limited number of clones may be sequenced and the posssible effects of diet and other environmental factors on the biodiversity and composition of the rumen microbial community may not be considered [3]. Based on human and animal (mouse, rat) studies, several candidate bacteria, such as *Clostridium* sp. [30], *Eubacterium* sp. [31, 32], *Bifidobacterium* sp. [33], *Ruminococcus* sp. or *Streptococcus* sp. [34], are commonly in the rumen [35] and may be involved in daidzein metabolism.

The aims of the present study were to compare the effects of two diets containing different isoflavone concentrations on the isoflavone transfer from feed into milk, and to identify possible relationships between rumen microbiota profiles and daidzein and equol levels.

## Materials and Methods

### Ethics Statement

Animal handling in this study was performed according to current Czech legislation (Act No. 246/1992 Coll. to protect animals against cruelty, as amended). Sampling techniques used in our study do not need special approval.

### Animals and diets

The on-farm experiment was carried out on twelve lactating Czech Fleckvieh x Holstein cows (average milk yield at the beginning of the trial: 23.6 ± 3.02 kg/d) that were divided into two groups with similar mean milk yield. Cows were fed individually, twice daily (6.30 and 16.30 h), and, *ad libitum*, with a diet based on maize silage (30 kg/d), meadow hay (2 kg/d), and a supplemental mixture (8 kg/d, all on an as-fed basis). The control group (CTRL) received the basal diet only, while the experimental group (EXP) received the basal diet supplemented with 12.5 g of 40% isoflavone extract (Biomedica, Prague, Czech Republic) at each feeding. The experiment lasted 14 days and consisted of a 12-day preliminary period followed by a 2-day collection period. Prior to the experiment, there was a 1-week period for animal adaptation to the diet.

### Sample collection

During the collection period, feed intake was recorded and samples of individual feedstuffs were taken to determine the chemical composition.

Cows were milked twice a day (7:00 and 17:00 h). Milk yield was recorded and milk samples were collected at each milking during the collection period. Each day, samples from evening and morning milking were mixed, proportional to milk yield, into one representative sample per cow. Samples for determining basic chemical composition were treated with the preservative 2-bromo-2-nitropropane-1.3-diol (Bronopol; D&F Control Systems, Inc. USA), and stored at 6°C until analysis. Samples for determining isoflavone content were stored at -20°C without any preservative until analyses.

Rumen fluid was collected on the last day of the collection period, 3 hours after the morning feeding, using flexible, stainless-steel stomach-tubes. Rumen fluid samples were placed in bottles with CO_2_, and were transferred to the laboratory in a heat-stable box. Immediately, pH was measured using an accurate pH-meter. Then, each fluid sample was mixed and filtered through four layers of cheesecloth. Samples of the fluid filtrates were collected to determine chemical composition and to identify bacteria.

### Sample analyses

Feedstuffs were evaluated using dry matter (DM) obtained by drying at 55°C for 24 h, followed by milling through a 1-mm screen and drying for another 4 h at 103°C. Crude protein (CP), crude fibre (CF), ash, and fat contents were estimated using methods that meet the standards of the Association of Analytical Communities [36]. Neutral detergent fibre (NDF, with α-amylase) content was estimated according to Vansoest’s method [37], and ash-free acid detergent fibre (ADF) was estimated according to a method by Goering and Van Soest [38].

Basic milk constituents were analysed using an infrared analyser (Bentley Instruments 2000, Bentley Instruments Inc., USA). Ammonia concentrations in the rumen fluid samples were determined using by the Conway microdiffusion method [39]. The concentrations of volatile fatty acids (VFAs) were measured using a CHROM-5 gas chromatograph (INGOS Ltd Laboratory Division, Prague, Czech Republic) fitted with a glass column packed with 80/120 Carbopack B-DA/4% CARBOWAX 20 M. Trimethylacetic acid was used as an internal standard and nitrogen was the carrier gas.

### Equol and Isoflavone Quantification

Isoflavones in feed were identified as previously described in the literature [40]. Briefly, homogenised feed samples were hydrolysed with 9.5 mol/l hydrochloric acid and ethanol under a reverse condenser at the boiling point of ethanol. After hydrolysis, the extracts were cleaned up using a solid phase extraction (SPE) procedure on Oasis HLB cartridges, (Waters, UK). The samples were subsequently analysed using high performance liquid chromatrography with diode-array detection (HPLC-DAD).

Samples of milk and rumen fluid were prepared as described previously [41]. In brief, 100 ng of the internal standard 4-HBP was added to each milk sample before deproteinization by mixing with acetone. The precipitate was removed by centrifugation and the acetone was evaporated. Each sample was then incubated with 100 μl of β-glucuronidase/sulphatase (*Helix pomatia*) in sodium acetate buffer (75 mM, pH 5) at 37°C overnight. Aglycons were extracted by shaking with ethyl acetate. The organic phase was evaporated to dryness, dissolved in 1 ml of methanol:water (1:1, v/v) and filtered through a 0.20μm filter. The samples were subsequently analysed by HPLC coupled with mass spectrometry-time of light (MS-(TOF)).

Rumen fluid samples were centrifuged for 30 min at 11 000×g. A total of 100 ng of internal standard 4-HBP was added to the supernatant of each sample, and the analytes were extracted twice in-to ethyl acetate by intensive shaking for 3 min. Subsequently, solvents were separated by centrifugation (5 min, 11 000×g). Organic fractions were collected and evaporated to dryness. The residues were thoroughly dissolved in 1 ml of methanol:water (1:1, v/v). Each sample was filtered through a 0.20μm filter and analysed by HPLC-MS.

HPLC analyses were performed using an Agilent Technologies 1260 system coupled with a time-of-light (TOF) mass spectrometer (Agilent Technologies 6224) equipped with a dual electrospray ionization (ESI) ion source operated in negative ionization mode. The on-line coupled HPLC system consisted of a h-ALS-SL G1367C wellplate sampler, a G1312B binary pump, a G1316C thermostated column compartment, and a VL+ G1315C diode array detector (Agilent Technologies). The drying gas temperature was set to 325°C, the drying gas flow to 10 l/min, the nebulizer pressure to 35 psi, the VCap voltage to 4000 V, the fragmentor voltage to 200 V, the skimmer1 voltage to 65 V, and the OctopoleRFPeak voltage to 750 V. Chromatographic separation was carried out on a Zorbax Extend-C18 column (2.1 × 50 mm, 1.8 μm, Agilent). The HPLC mobile phases 0.1% acetic acid in water and methanol with gradient elution at a flow-rate of 0.2 ml/min were used [41].

### DNA extraction and Real-Time PCR

One millilitre of each rumen fluid sample was centrifuged at 10 000xg for 20 min at 4°C. Consequently, the pellets were used for DNA isolation using a BiOstic^®^ Bacteremia DNA Isolation Kit (MoBio) according to the manufacturer’s instructions. The DNA concentration and purity were evaluated by optical density using a BioWave nano-spectrophotometer (Implen, DE) at wavelengths of 230, 260, and 280 nm, and DNA was stored at -20°C until use.

Real-time PCR was carried out using the KAPA SYBR FAST qPCR Master Mix (KapaBiosystems) in a LightCycler 480 thermocycler (Roche). Primers flanking the V3/V4 hypervariable region were used to quantify total bacteria. PCR was initiated with a hot start for 3 min at 95°C followed by 35 cycles of 15 sec at 95°C, 30 sec at 55°C. Melting temperatures were determined after the PCR to verify the correctness of each PCR product. The concentrations were calculated from Ct values using individual quantification standards prepared by dilutions of pGEM-T Easy plasmids with cloned PCR products.

### PCR amplification of the V3/V4 region of bacterial 16S rRNA genes

DNA extracted from the rumen fluids was used as a template in PCRs with the forward primer 5' TCGTCGGCAGCGTCAGATGTGTATAAGAGACAG-CCTACGGGNGGCWGCAG 3', and reverse primer 5' GTCTCGTGGGCTCGGAGATGTGTATAAGAGACAG-GACTACHVGGGTATCTAATCC 3' flanking the V3/V4 hypervariable region mostly used in next generation sequencing (NGS)-based diversity studies [42], including the underlined Illumina NEXTERA adapter overhang nucleotide sequences. Amplification was done in a 25 μl volume using KAPA HiFi HotStart ReadyMix (KapaBiosystems), 0.3 μM primers, and 2.5 μl of isolated DNA, with cycling conditions consisting of a hot start at 95°C for 3 min, followed by 30 cycles of incubation at 95°C for 30 s, 55°C for 30 s, and 72°C for 30 s. PCR was terminated by a final extension at 72°C for 5 min. After PCR, the amplification products were precipitated using a mixture of 30% PEG8000/30mM MgCl_2,_ centrifuged for 15 min at 10 000xg at room temperature, and dissolved in 15 μl of 1xTE buffer. Consequently, 5 μl each of the purified PCR samples was tagged with NEXTERA indexes using the Nextera XT Indexes Kit (Illumina, USA) and the KAPA HiFi HotStart ReadyMix (KapaBiosystems) in a 25 μl reaction volume according to the Illumina protocol. PCR products were purified using a GeneRead DNA size selection Kit (Qiagen, Germany) according to the manufacturer’s protocol with elution to 17 μl of 1xTE buffer, quantified using the Kapa Library Quantification Kit (Kappa Biosystems), and diluted to 4 nM concentrations. The final library was subjected to NGS performed using a MiSeq^®^ Reagent Kit v3 (600 cycle) and an Illumina MiSeq sequencer according to the manufacturer’s instructions (Illumina, USA).

### Sequences analyses

Mothur v1.25 [43] was used for sequence analysis, OTU detection, taxonomic assignment, and phylogenetic analysis. Weighted UniFrac calculations [44] and principle component analysis, using the Jaccard index, were carried out with Phyloseq [45] to compare bacterial populations among different samples. The sequence data was first subjected to quality control, including removal of: all sequences shorter than 400 bp, sequences with mismatches in the barcode region, sequences containing more than ten non-standard bases, and sequences with a homopolymer length greater than 8. The remaining sequences were aligned using the Silva bacterial database [46]. After the alignment, the ends of the sequences were optimized using the filter.seqs command in Mothur. Chimeric sequences were detected and removed using the sequence collection (UCHIME) as its own reference dataset [47]. Sequences were then subsampled to obtain a uniform number of sequences per sample for all subsequent analyses. A distance matrix was constructed using Mothur at phylogenetic distances of 0.03 (species), 0.07 (genus), and 0.25 (phylum), respectively, to define OTUs. Consequently, Phyloseq was used to: (1) calculate sequence coverage, species diversity using inverse Simpson and Shannon-Weiner indices and the Chao index, and (2) to define the core microbiome in samples. The Mann-Whitney non-parametric test was used to examine the significance of differences in the abundance of OTUs in the samples. Differences with a P-value <0.05 were determined to be statistically significant.

### Calculations and statistical analysis

Isoflavone transfer from feed into milk was expressed as the carry-over rate for either individual or total isoflavones based on previous studies by Steinshamn et al. [19], and Flachowsky et al. [48], respectively, according to the following formulas:

$$\text{Carry}-\text{over of daidzein }\left[\mu \text{g}/\text{mg}\right]=\frac{\text{sum of daidzein and equol excreted in milk}}{\text{sum of daidzein intake}}$$

$$\text{Carry}-\text{over of genistein }\left[\mu \text{g}/\text{mg}\right]=\frac{\text{sum of genistein excreted in milk}}{\text{sum of genistein intake}}$$

$$\text{Carry}-\text{over of glycitein }\left[\mu \text{g}/\text{mg}\right]=\frac{\text{sum of glycitein excreted in milk}}{\text{sum of glycitein intake}}$$

$$\text{Carry}-\text{over of total isoflavones }\left[\mu \text{g}/\text{mg}\right]=\frac{\text{sum of isoflavones excreted in milk}}{\text{sum of isoflavones intake}}$$

Data concerning nutrient intake, milk yield, and isoflavone concentrations obtained in the experiment were analysed using the general linear models (GLM) procedure of the Statgraphics 7.0 package (Manugistics Inc. and Statistical Graphics Corporation, Rockville, Maryland, USA). One-way ANOVA was used to determine differences in rumen parameters, and multifactor ANOVA with treatment, cow, and day of sampling effects was used to determine differences in dietary and milk parameters.

The Mann-Whitney non-parametric test was used to examine the significance of differences in the abundance of the bacteria and OTUs between experimental and control samples. Differences with a P-value <0.05 were considered statistically significant. The correlation coefficient was calculated and used to assess the correlation between representation of the OTUs and daidzein concentration by applying the CORREL function in Microsoft Office Excel 2007.

## Results

### Nutrient intake, milk yield, milk composition, and isoflavone carry-over rate

The average daily intake of nutrients and isoflavones is presented in Table 1 and S2 Table. The extract did not influence the palatability of the diet. Cows consumed all their diets, respective refusals were monitored daily and did not exceed 5% of the diet and consisted mainly of roughage.

**Table 1:** Isoflavone levels in feed, rumen fluid, and milk, isoflavone yield and carry-over. Average daily isoflavone intakes, rumen fluid isoflavone concentrations, milk isoflavone concentrations, isoflavone yields, and isoflavone carry-overs of dairy cows fed a basal diet either unsupplemented (CTRL) or supplemented with 40% isoflavone extract (EXP).

Intake of:		CTRL	EXP	SEM	P
Daidzein	mg/d	3,405.75	1,0617.60	104.689	<0.001
Genistein	mg/d	4,383.43	4,596.89	132.687	0.268
Glycitein	mg/d	611.94	792.07	15.796	<0.001
Total isoflavones	mg/d	8,401.12	16,006.60	253.057	<0.001
**Rumen fluid concentrations of:**					
Daidzein	μg/L	21.06	119.99	19.616	0.005
Glycitein	μg/L	0.08	0.71	0.492	0.397
Genistein	μg/L	0.52	0.97	0.218	0.169
Equol	μg/L	337.91	662.55	65.718	0.006
Total isoflavones	μg/L	359.67	784.33	73.749	0.002
**Milk concentrations of:**					
Daidzein	μg/L	11.78	47.85	5.354	<0.001
Genistein	μg/L	4.63	9.99	1.827	0.051
Glycitein	μg/L	2.69	31.94	11.114	0.077
Equol	μg/L	77.78	186.30	19.29	<0.001
Total isoflavones	μg/L	96.89	276.07	25.376	<0.001
Isoflavone yield					
Daidzein	μg/d	286.50	1,094.14	134.24	<0.001
Genistein	μg/d	113.58	230.67	44.086	0.074
Glycitein	μg/d	64.67	742.36	261.454	0.081
Equol	μg/d	1,931.13	4,235.89	463.607	0.002
Total isoflavones	μg/d	2,395.89	6,303.06	640.388	<0.001
Carry-over rate of:					
Daidzein	μg/mg	0.65	0.50	0.067	0.122
Genistein	μg/mg	0.03	0.05	0.01	0.095
Glycitein	μg/mg	0.11	0.94	0.336	0.094
Total isoflavones	μg/mg	0.29	0.39	0.043	0.100

Intake of dry matter and other nutrients did not differ between groups (P>0.05), however CTRL cows tended to have higher intake of dry matter in comparison to EXP cows (P = 0.090). Individual feedstuff contents contained relatively high isoflavone concentrations with the supplemental mixture being the richest feedstuff source resulting in average daily isoflavone intake by the CTRL group of 8,401.12 mg/d. The 40% soybean extract used in our study contained a high proportion of daidzein and low proportions of genistein and glycitein. These isoflavone proportions reflect the influences of geographical origin on isoflavone concentration, as proven in many studies [21, 49], as well as on the daidzein, genistein, and glycitein ratios [48]. Supplementation of the basal diet with 40% soybean extract increased the average daily intake of isoflavones in the EXP group nearly twofold (16,006.55 mg/d, P<0.001). The extract used in our study contained a high daizein proportion (306.7 g/kg) and low proportions of genistein and glycitein (18.8 and 8.8 g/kg, respectively). Milk yield and composition are provided in S3 Table. CTRL cows demonstrated a trend towards a higher milk yield than EXP cows (P = 0.090), probably due to similar trend in dry matter intake. The concentrations of basic milk constituents did not differ significantly between the groups (P>0.05).

Daidzein, genistein, glycitein, and equol were detected in milk from both groups (Table 1). Genistein and glycitein concentrations tended to be higher in EXP cows compared to CTRL cows (P = 0.051 and 0.077, respectively), while daidzein and equol concentrations increased from 11.78 to 47.85 μg/L and from 77.78 to 186.30 μg/L, respectively (P<0.001). These increases resulted in a total isoflavone concentration that was nearly three times higher in EXP milk (276.07 μg/L) than in CTRL milk (96.89 μg/L, P<0.001). The total isoflavone transfer from feed into milk was low in both the CTRL and EXP groups with values of 0.29 and 0.39 μg/mg, respectively (P>0.05).

### Analysis of rumen bacterial content

The basic characteristics of the rumen milieu and the isoflavone content in rumen fluid are provided in S4 Table. The pH and ammonia content were not affected by the supplemental treatment (P>0.05). Individual (except for butyric acid) and total VFA contents were higher in CTRL cows compared to EXP cows (P<0.05). However, the rumen milieu characteristics of both groups were within normal physiological ranges. Glycitein and genistein concentrations in rumen fluid collected 3 h after feeding were very low and did not differ between groups (P>0.05), while daidzein and equol concentrations were higher in EXP cows compared to CTRL cows (P<0.01). Total isoflavone concentrations in EXP cows was more than two times higher than in CTRL cows (784.33 and 359.67 μg/L, respectively (P<0.01)). There are no comparable studies describing the influence of isoflavone intake on isoflavone concentrations in the rumen fluid.

**Fig 1:**
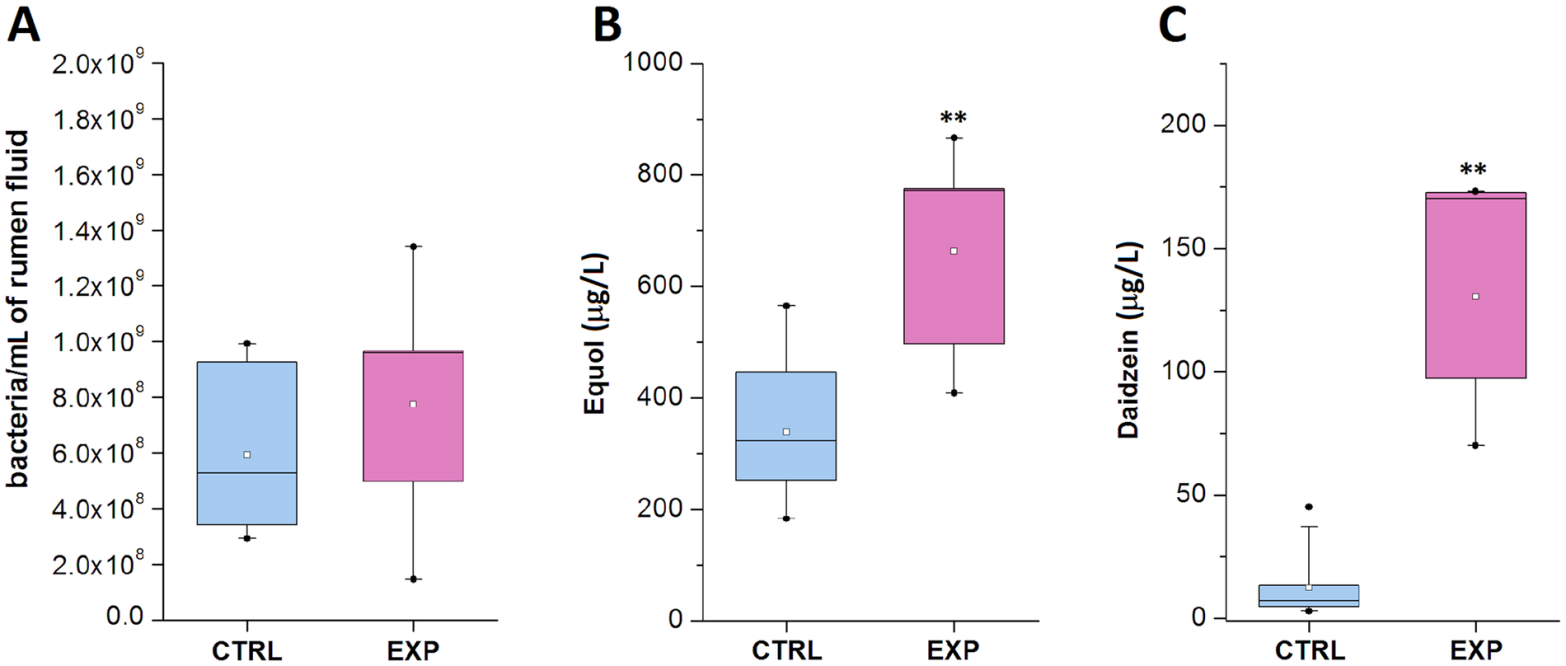
Characterization of rumen fluid of experimental (EXP) and control (CTRL) groups. (A) Amount of total bacteria in 1 ml of rumen fluid from control (CTRL) and experimental (EXP) cows was quantified using qPCR according to the average 16S rRNA copy number per bacterial genome. (B, C) Daidzein and equol concentrations in rumen fluid were quantified using HPLC coupled with MS-(TOF). Control cows, n = 6; experimental cows, n = 6. Open squares indicate minima and maxima, whiskers indicate the 5th and 95th quantile, and boxes indicate the 25th and 75th quantile. A horizontal line indicates the median, and a square indicates the mean (** P < 0.05, control group versus experimental group).

The cow rumen microbiome plays an important role in animal health and productivity and is a very dynamic system that changes with age [50] or diet [51]. The qPCR method was adapted to observe the impact of isoflavone supplementation on the content of total rumen bacteria. Primers that were previously tested with 16S rRNA and that produce approximately the same DNA product length were used. Average bacterial 16S rRNA copy numbers (4.3) from previous publications were used to avoid the impact of 16S rRNA gene variability in bacterial genomes on the analysis of the bacterial community [52]. The analysis of total bacteria content did not reveal any significant changes between the control and experimental groups (Fig 1A).

### 16S rRNA sequencing revealed differences between the control and experimental groups

**Fig 2:**
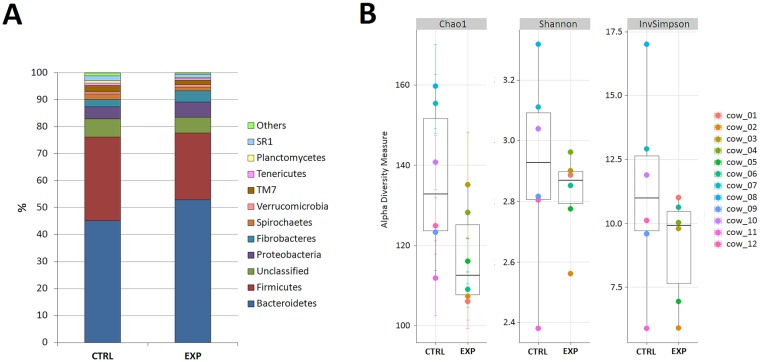
Diversity of bacterial phyla (A) and species richness (B) observed in the control (CTRL) and experimental (EXP) groups. (A) The percentage of total reads (x-axis) assigned to each phylum is plotted for CTRL and EXP (y-axis). (B) Species richness of CTRL and EXP rumen samples included in this study at 97% similarity. Chao1 panel shows estimated species richness, and species diversity is represented by Shannon and InvSimpson indices.

The V1-V3 region of 16s rDNA gene was sequenced on MiSeq sequencer using a MiSeq Reagent Kit v3 (Illumina, USA) to further examine the detailed composition of the rumen microbiomes of experimental and control cows. Sequencing produced 1,322,929 reads that passed quality control filters (S5 Table). The sequences were subsampled (n = 30,984) to ensure that a consistent and equal number of sequences from each cow were used for further analysis. The experimental cows had fewer observed OTUs at a 0.03 distance level, and a lower Chao1 index than control cows (Student t-test, P = 0.04). The experimental cows also exhibited reduced microbial richness compared to the control cows based on the calculated Inverse Simpson and Shannon indices (Fig 2). Diversity was noticeably higher in the observed OTUs from control cows compared to OTUs from experimental cows. In total, 21 phyla, 76 orders, 146 families, and 321 bacterial genera were identified in the samples from all the animals (S6 Table).

Similar to rumen microbiomes that have been analyzed to date, the predominant phyla in all samples were *Bacteroidetes* (~55% of total bacteria) and *Firmicutes* (~5% of total bacteria). However, the *Bacteroidetes* to *Firmicutes* ratio and the proportion of sequences varied between the experimental and control groups. *Bacteroidetes* were more prevalent in the experimental group (P = 0.01), changing the *Bacteroidetes* to *Firmicutes* ratio from 1.49 to 2.64 in that group (Fig 2). Compared to the control group, the experimental group had significantly lower levels of *Planctomycetes* (P = 0.01) and relatively higher levels of *Fibrobacteres*, showing high variability in the relative amounts in each sample (Fig 2).

### Comparison of bacterial communities between groups

**Fig 3:**
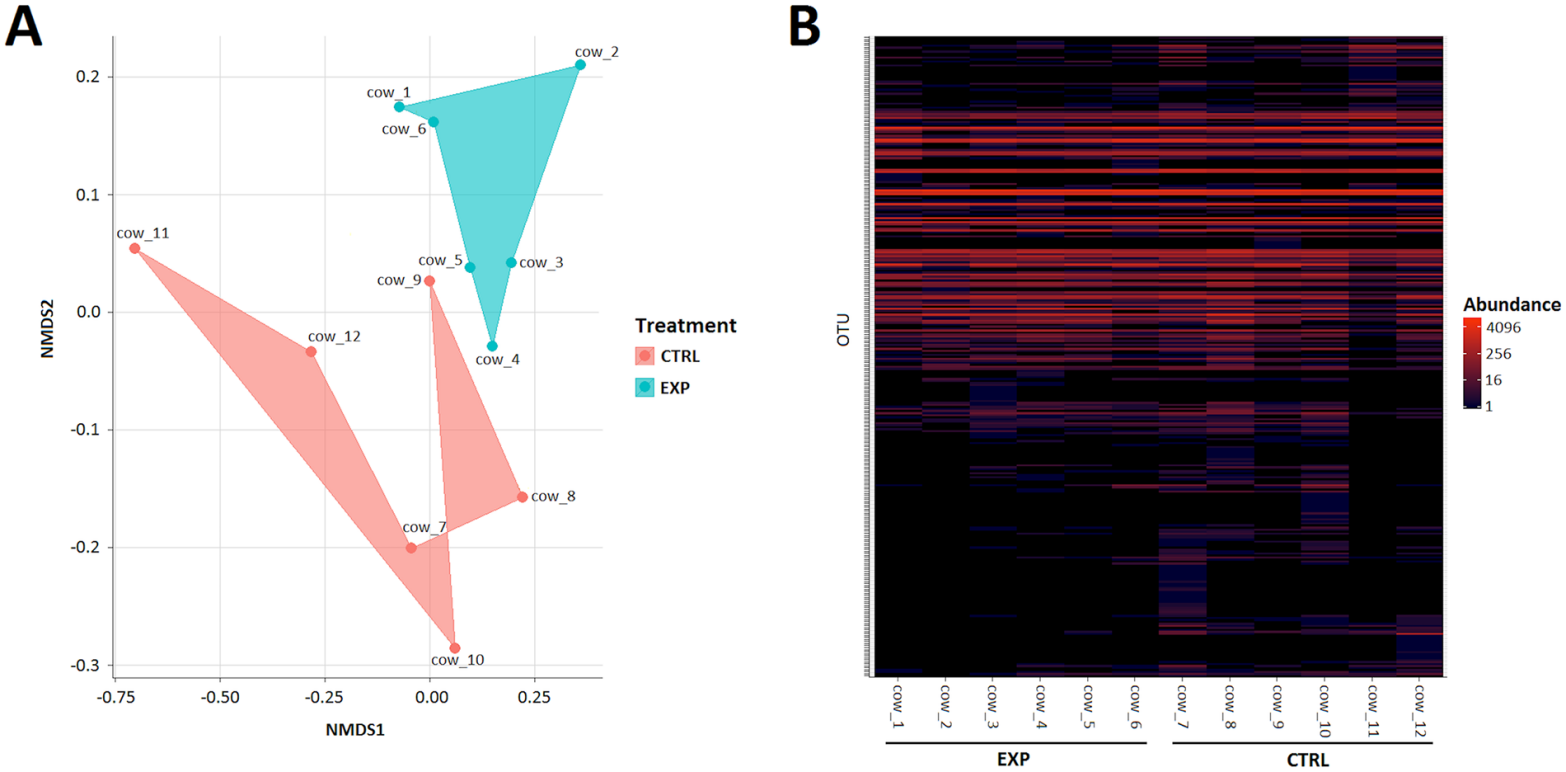
Non-metric multidimensional scaling (NMDS) plot (A) and heat map representation (B) of the 200 most abundant bacteria taxa across control (CTRL) and experimental (EXP) rumen samples. (A) NMDS plot was based on Jaccard's index; rumen microbial communities of each group are indicated by different colours: blue circles =  experimental group; red circles = control group. (B) Heat map, based on NMDS ordination on the Jaccard distance, showing abundance of the 200 most abundant OTUs at a distance of 0.03 across the CTRL and EXP rumen samples.

A non-metric multidimensional scaling (NMDS) plot at 97% identity, based on a Jaccard similarity, clearly showed that experimental cows clustered separately from control cows (Fig 3A). In addition, weighted UniFrac analysis confirmed that the phylogenetic lineages were significantly different between the tested groups (WScore = 0.50, P<0.001). A corresponding heatmap, based on an ecology-oriented variant of the HeatMap approach in phyloseq, showed that the difference between the groups was based mainly on low abundance taxa (Fig 3B).

**Fig 4:**
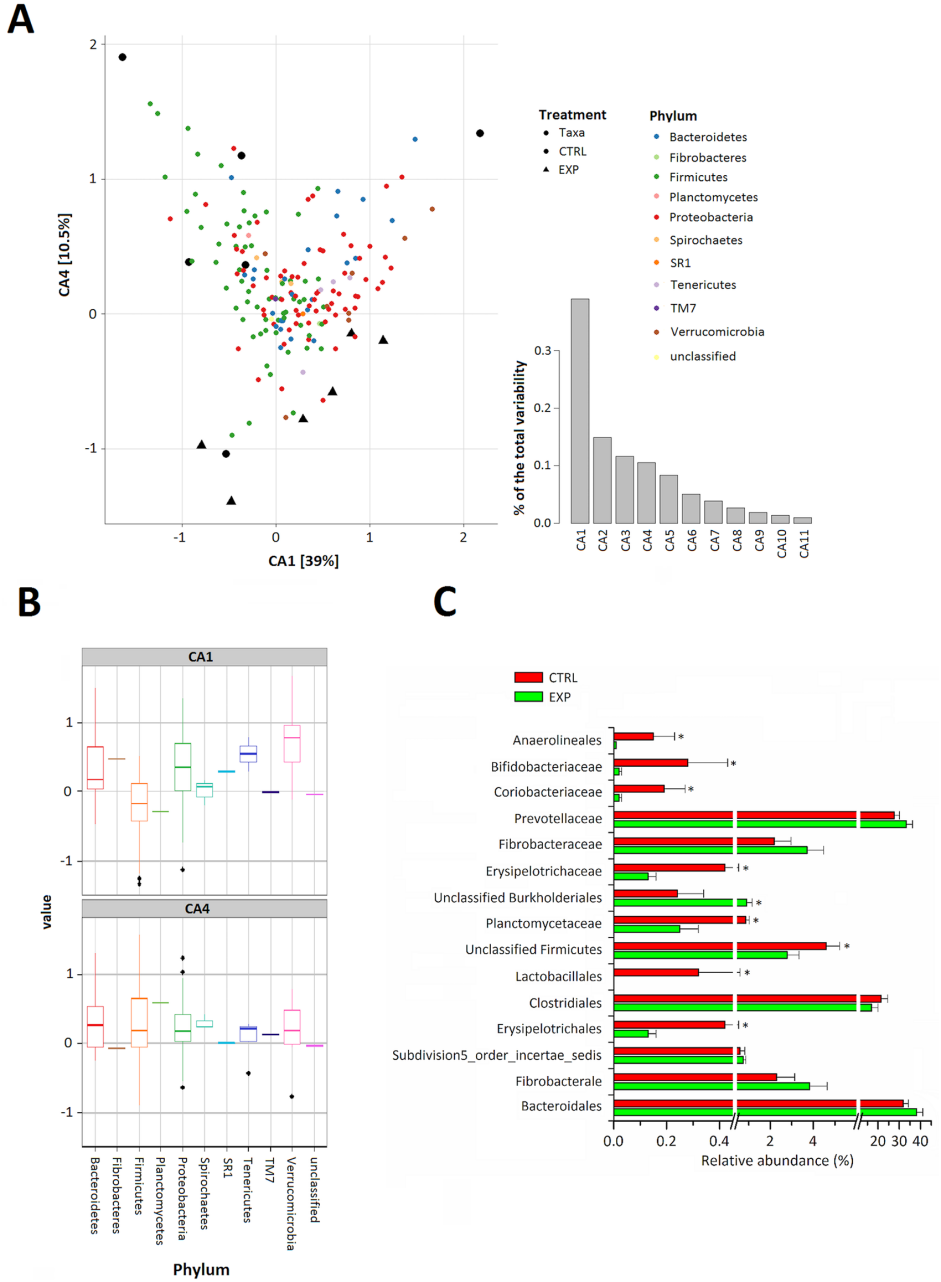
A comparison of rumen microbiota between experimental and control groups. (A) BiPlot Correspondence Analysis (CA) of rumen microbiota showing separation of experimental and control group samples on the first (CA1) and fourth axes (CA4) which are responsible for 50% of the the total (chi-square) variability. (B) Boxplots of the ten most strongly represented phyla conveying information about the taxa pattern that contributes to the separation of experimental cows from control ones. (C) The relative abundance (%) of differentially abundant families and genus. Control cows, n = 6; experimental cows, n = 6. Data are presented as the mean ± SEM (* P < 0.05, control group versus experimental group).

Correspondence analysis (CA) was used to further identify differences between the experimental and control groups. Scree plot showed that the first four CA axes accounted for more than 75% of the total variability with separation between the experimental and control groups occurring mainly on the first and fourth axes (CA1 and CA4; Fig 4A). Variability of the bacterial communities within the groups was noticeably high and corresponded to the high diversity in the OTUs. A sequence comparison of the ten most abundant phyla showed that the major contributors to the separation between the experimental and control groups were the high representations of bacteria belonging to *Bacteroidetes*, *Proteobacteria*, *Fibrobacteres*, and *Verrucomicrobia*, and the reduced abundance of bacteria belonging to *Firmicutes* and *Planctomycetes* (Fig 4B).

*Bacteroidales*, *Fibrobacterales*, *Subdivision5_order_incertae_sedis*, and *Burkholderiales* were the predominant orders, respectively, in phyla that were more abundantly represented in experimental cows. A subsequent, deeper phylogenetic classification of the reads revealed that the major overrepresented families in experimental samples were *Prevotella*, *Fibrobacteraceae*, and unclassified *Burkholderiales* (Fig 4C). Within *Firmicutes*, orders such as *Clostridiales*, *Erysipelotrichales*, and *Lactobacillales* were all found to be more abundant in control cows than in the experimental cows. Moreover, the control and experimental cows exhibited different proportions of minor families *Planctomycetaceae* (0.86% vs. 0.25%), *Anaerolineales* (0.15% vs. 0.01%), *Coriobacteriaceae* (0.19% vs. 0.02), *Bifidobacteriaceae* (0.28% vs. 0.02%), and *Cardiobacteriaceae* (0.03% vs. 0.00%; Fig 4C).

### Correlation of bacterial communities with isoflavones levels

Correlation analysis was used to quantify the direction and statistical significance of the associations between orders or families of bacterial species and daidzein (representing the major compound in isoflavone extract) in collected rumen samples. Surprisingly, statistically significant positive correlations with daidzein were found only in orders *Burkholderiales* (r = 0.65, P = 0.02) and unclassified *Betaproteobacteria* (r = 0.58, P = 0.05). Within the *Firmicutes* order, negative correlations between daidzein and the families *Ruminococcaceae* (r = -0.68, P = 0.01), *Erysipelotrichaceae* (r = -0.60, P = 0.04), and *Eubacteriaceae* (r = -0.58, P = 0.05) were found. The analysis also indicated negative correlations between daidzein and *Planctomycetaceae* (*Planctomycetes*, r = -0.72, P = 0.008), *Coriobacteriaceae* (*Actinobacteria*, r = -0.59, P = 0.04), Armatimonadetes_gp2_family_incetae_sedis (*Armatimonadetes*, r = -0.62, P = 0.03), and unclassified *Actinomycetales* (*Actinobacteria*, r = -0.61, P = 0.03).

## Discussion

Although milk yield did not differ significantly between groups there was a tendency to higher milk production in CTRL cows compared to EXP cows (P = 0.090). This is in disagreement with other studies where soybean products were used as a source of isoflavones in dairy diets [40, 48, 53]. However in those studies dry matter intake in experimental group was higher or the same as in the control group while in our study CTRL cows tended to have higher intake of dry matter in comparison to EXP cows (P = 0.090). Because strong relationship exists between dry matter intake and milk production (R^2^ = 0.87, [54]), the difference in milk yield observed in our study was likely caused by difference in dry matter intake. However negative effect of isoflavone supplementation on fiber digestion in the rumen should also be considered.

Overall daidzein concentrations observed in both groups in this study were relatively high compared to those observed by Flachowsky et al. [48] or Třináctý et al. [40]. The comparison suggests that bacterial conversion of daidzein into equol in the rumen is inefficient when daidzein intake is high. Furthermore, differences in the ability of the rumen to degrade various isoflavone sources, as suggested by Křížová et al.[53], may also contribute to differences in the carry-over rates reported in the literature. In contrast to Flachowsky et al. [48] or Třináctý et al. [40] genistein concentrations in milk were very low, amounting to only 5% of total isoflavones.

Equol concentrations in CTRL cows’ milk were similar to those reported by Flachowsky et al. [48] or Třináctý et al. [40], but higher than those reported by Antignac et al. [55]. Antignac et al. characterised milk from conventional farms that use common soybean components in the cows’ diets. After supplementation with 40% soybean isoflavone extract in our study, average equol concentration in milk increased to 186.30 μg/L from 77.78 μg/L. No comparable data concerning to soybean-derived dietary isoflavones exist, but similar equol concentrations values in milk can be obtained after feeding cows with grass clover, white clover, or white clover sillages in some studies [19, 56]. However, much higher equol concentrations in milk have been reported in the literature after feeding red clover or red clover silage-based diets ranging from 272 μg/kg up to 1,490 μg/kg [18, 19, 57–59].

In our study, total isoflavone transfer from feed into milk was comparable to that of Flachowsky et al. [48]. However, the latter study showed a decline in the carry-over rate with increases in isoflavone intake. Our study showed the oposite trend. The carry-over rates for daidzein and equol in both groups in our study were considerably lower than those determined by Třináctý et al. [40], Flachowsky et al. [48] or Křížová et al.[53]. This discrepancy was probably caused by differences in isoflavone intake that were several times greater than in the above-mentioned studies. Similar trends were observed for the carry-over rate of genistein.

The VFA produced by microbial fermentation in the rumen are the main source of energy for ruminants. Thus, the amount and profile of VFA formed in the rumen has consequences for the efficiency of energy utilization, production of methane, risks of ruminal acidosis and composition of animal products. While total production of VFA closely depends on the amount of the intake of rumen fermentable organic matter VFA profiles can be affected by dry matter intake, amount of digestible organic matter and digestible NDF and rumen starch digestibility. Furthermore, other factors such as particle size or buffer supply may contribute to variability in VFA profiles [60]. Studies focused on the impact of isoflavones on the rumen milieu are scarce and inconsistent. In our study, the CTRL group possessed more individual (except for butyric acid) and total VFA content than the EXP group (P<0.05), however, the proportion of propionic acid in total VFA content was similar in both groups. Zhu et al. (2002) demonstrated the direct effect of daidzein on rumen microbial activity using *in vitro* techniques, with goat rumen fluid as the inoculum. Contrary to our findings Zhu et al. showed that daidzein at concentrations of 5 and 10 mg/l significantly increased the proportion of propionate in total VFA content. However, daidzein at higher concentrations (above 20 mg/l) did not show significant effects on rumen microbial fermentation. Furthermore, they mentioned that the effect of 10 mg/l of daidzein on VFA profiles became evident only after one hour incubation. They suggested that daidzein may cause the biphasic response, like estrogenic and anti-estrogenic effects that are observed at low or high isoflavone concentrations, respectively.

As documented in many studies (e.g. Woclawek-Potoczka et al.), [61] isoflavones and their metabolites are present in circulating blood after absorption. These circulating isoflavones and their metabolites in the blood affect the rumen microorganisms in addition to the initial direct effect of isoflavones within the rumen [62]. Other factors such as the fluctuation of blood testosterone levels that can enter the rumen via saliva or via the rumen epithelium may also affect VFA content and rumen bacterial protein [63, 64]. Further studies are needed to clarify these observations.

Rumen microflora play a key role in the metabolism and bioavailability of isoflavones in ruminants. In recent years, there have been attempts to isolate bacterial strains that can metabolize isoflavones. Some of the strains were isolated from human faeces [34, 65–67] 2005) or rumen fluid [25].

However, studies focused on isoflavone effects on the composition of the rumen microbial population are scarce. When studying the effect of daidzein supplementation on microbial composition of the goat rumen Yao *et al*. [68] detected shifts in rumen microbiota composition. They found that most of the denaturing gradient gel electrophoresis (DGGE) bands were common to both control and daidzein-supplemented rumen samples. While some bands were enriched, the others disappeared after daidzein supplementation, suggesting that daidzein influences the rumen microbial composition.

Clavel et al. [69], in their trial on postmenopausal women, proved that isoflavones altered the dominant microbiota, both quantitatively and qualitatively. They found that microbial patterns were 73% similar to day 0 after 1 month of isoflavone supplementation, reflecting changes in the distribution of dominant bacterial species compared to characteristic intraindividual stabilities ilustrated by 83–96% similarity over a period of 3 months [70]. In general, our study produced similar findings.

Furthermore, Decroos et al. [67] isolated a mixed bacterial culture from human fecal samples that could transform daidzein into equol, and identified *Lactobacillus mucosae* EP12, *Enterococcus faecium* EPI1, and *Finegoldia magna* EPI3 as bacteria involved in isoflavone metabolism. However, these three species could not produce equol from daidzein in the pure culture, suggesting that some uncultured or undetectable bacterial species were responsible for equol production.

In our study, we determined that the *Bacteroidetes* phylum represented about 55% of total bacteria and *Firmicutes* phylum represented about 5% of total bacteria in the rumen microbiomes of all animals tested. Our findings agree with those of De Boever et al. [71] who isolated several major bacterial groups, including *Bacteroides spp*. and *Bifidobacterium spp*., involved in isoflavone metabolism in their colonic model. Similarly, according to Menon et al. [72] *Bacteroidetes*, *Firmicutes*, and *Proteobacteria* were the most abundant phylotypes in the gastrointestinal tracts of mice, regardless of the diet consumed or genotype.

## Supporting Information

S1 Table Composition of supplemental mixture. (PDF)

S2 Table Average daily intake of nutrients and isoflavones of dairy cows fed basal diet either unsupplemented (CTRL) or supplemented with 40% isoflavone extract (EXP). (PDF)

S3 Table Effects of supplementation of basal diet (CTRL) with 40% isoflavone extract (EXP) on average milk yield, milk contents, yield of milk components and isoflavones, and isoflavone carry-over rate from feed to milk. (PDF)

S4 Table Effect of supplementation of basal diet (CTRL) with 40% isoflavone extract (EXP) on characteristics of the rumen milieu of dairy cows. (PDF)

S5 Table Workflow of sequence analysis. (PDF)

S6 Table OTU table of all microbiota detected in rumen of control and experimental cows classified down to genus level. (XLSX)
