# Supplementary material for: Effects of Isoflavone-Enriched Feed on the Rumen Microbiota in Dairy Cows
Source: PLoS One. 2016 Apr 28;11(4):e0154642. doi: 10.1371/journal.pone.0154642 (PMC4849651; doi:10.1371/journal.pone.0154642)
Supplement: S1 Table — (PDF) [file pone.0154642.s001.pdf]

Table S1. Composition of supplemental mixture.

Wheat	%	27.6
Barley	%	10.0
Maize grain	%	15.0
Soybean extraction meal, peeled, toasted	%	19.0
Rapeseed extraction meal	%	11.0
Malt sprouts	%	7.6
Schaumann energy	%	3.3
Rindamin Gim Spezial	%	2.3
SME Bovi Top (microminerals and vitamines mixture)	%	0.5
Sodium bicarbonate (NaHCO3)	%	1.1
Monocalcium phosphate ($Ca(H_2PO_4)_2$)	%	0.5
Magnezit ($MgCO_3$)	%	0.2
Sodium chloride (NaCl)	%	0.3
Limestone (CaCO3)	%	1.6
