# Supplementary material for: Effects of Isoflavone-Enriched Feed on the Rumen Microbiota in Dairy Cows
Source: PLoS One. 2016 Apr 28;11(4):e0154642. doi: 10.1371/journal.pone.0154642 (PMC4849651; doi:10.1371/journal.pone.0154642)
Supplement: S2 Table — (PDF) [file pone.0154642.s002.pdf]

Table S2. Average daily intake of nutrients and isoflavones of dairy cows fed basal diet either unsupplemented (CTRL) or supplemented with 40% isoflavone extract (EXP)**.**

Intake of:		CTRL	EXP	SEM	P
Dry matter	kg/d	22.36	21.30	0.419	0.090
Crude protein	kg/d	3.69	3.51	0.084	0.132
Crude fiber	kg/d	4.51	4.31	0.075	0.071
NDF[1]	kg/d	8.78	8.38	0.148	0.070
ADF[2]	kg/d	5.00	4.77	0.083	0.072
Daidzein	mg/d	3,405.75	10,617.60	104.689	<0.001
Genistein	mg/d	4,383.43	4,596.89	132.687	0.268
Glycitein	mg/d	611.94	792.07	15.796	<0.001
Total isoflavones	mg/d	8,401.12	16,006.55	253.057	<0.001

[1] neutral detergent fiber
[2] acid detergent fiber
