# Supplementary material for: Effects of Isoflavone-Enriched Feed on the Rumen Microbiota in Dairy Cows
Source: PLoS One. 2016 Apr 28;11(4):e0154642. doi: 10.1371/journal.pone.0154642 (PMC4849651; doi:10.1371/journal.pone.0154642)
Supplement: S3 Table — (PDF) [file pone.0154642.s003.pdf]

Table S3. Effects of supplementation of basal diet (CTRL) with 40% isoflavone extract (EXP) on average milk yield, milk contents, yield of milk components and isoflavones, and isoflavone carry-over rate from feed to milk.

		CTRL	EXP	SEM	P
Milk yield	kg/d	25.08	22.36	1.084	0.090
Fat	g/100 g	6.19	5.98	0.277	0.600
Protein	g/100 g	2.95	2.93	0.092	0.847
Lactose	g/100 g	4.60	4.56	0.048	0.629
Urea	mg/100mL	27.42	29.92	1.545	0.265
Daidzein	µg/L	11.78	47.85	5.354	<0.001
Genistein	µg/L	4.63	9.99	1.827	0.051
Glycitein	µg/L	2.69	31.94	11.114	0.077
Equol	µg/L	77.78	186.30	19.290	<0.001
Total isoflavones	µg/L	96.89	276.07	25.376	<0.001
Isoflavone yield					
Daidzein	µg/d	286.50	1094.14	134.240	<0.001
Genistein	µg/d	113.58	230.67	44.086	0.074
Glycitein	µg/d	64.67	742.36	261.454	0.081
Equol	µg/d	1931.13	4235.89	463.607	0.002
Total isoflavones	µg/d	2395.89	6303.06	640.388	<0.001
Carry-over rate of:					
Daidzein	µg/mg	0.65	0.50	0.067	0.122
Genistein	µg/mg	0.03	0.05	0.010	0.095
Glycitein	µg/mg	0.11	0.94	0.336	0.094
Total isoflavones	µg/mg	0.29	0.39	0.043	0.100
