# Supplementary material for: Effects of Isoflavone-Enriched Feed on the Rumen Microbiota in Dairy Cows
Source: PLoS One. 2016 Apr 28;11(4):e0154642. doi: 10.1371/journal.pone.0154642 (PMC4849651; doi:10.1371/journal.pone.0154642)
Supplement: S4 Table — (PDF) [file pone.0154642.s004.pdf]

Table S4. Effect of supplementation of basal diet (CTRL) with 40% isoflavone extract (EXP) on characteristics of the rumen milieu of dairy cows.

		CTRL	EXP	SEM	P
pH		6.56	6.77	0.081	0.098
Ammonia	g/L	0.07	0.06	0.012	0.055
Acetic acid	g/L	1.89	1.56	0.044	<0.001
Propionic acid	g/L	0.84	0.70	0.028	0.005
Butyric acid	g/L	0.74	0.64	0.039	0.079
Total VFA[1]	g/L	3.64	3.05	0.098	0.002
Daidzein	µg/L	21.06	119.99	19.616	0.005
Glycitein	µg/L	0.08	0.71	0.492	0.397
Genistein	µg/L	0.52	0.97	0.218	0.169
Equol	µg/L	337.91	662.55	65.718	0.006
Total isoflavones	µg/L	359.67	784.33	73.749	0.002

[1] Total volatile fatty acids (VFA) includes also minor VFA (i-propionic, i-butyric, i-valeric, and valeric acids)
