# Supplementary material for: Effects of Isoflavone-Enriched Feed on the Rumen Microbiota in Dairy Cows
Source: PLoS One. 2016 Apr 28;11(4):e0154642. doi: 10.1371/journal.pone.0154642 (PMC4849651; doi:10.1371/journal.pone.0154642)
Supplement: S5 Table — (PDF) [file pone.0154642.s005.pdf]

Table S5. Workflow of sequence analysis.

Number of original reads	1322929
Number of sequences after removal ofall sequences shorter than 400 bp, sequences with mismatches in the barcode region and sequences containing more than ten non-standard bases	658806
Number of unique sequences	426429
Number of sequences after using the commands filter.seqs and pre.cluster (diffs=3)	154056
Number of sequences after the chimer and undesirables removal	85430
Number of sequences per sample after subsampling	30984
